# Prenatal Diagnosis for Primary Immunodeficiency Disorders—An Overview of the Indian Scenario

**DOI:** 10.3389/fimmu.2020.612316

**Published:** 2020-12-07

**Authors:** Reetika Malik Yadav, Maya Gupta, Aparna Dalvi, Umair Ahmed Bargir, Gouri Hule, Snehal Shabrish, Jahnavi Aluri, Manasi Kulkarni, Priyanka Kambli, Ramya Uppuluri, Suresh Seshadri, Sujatha Jagadeesh, Beena Suresh, Jayarekha Raja, Prasad Taur, Sivasankar Malaischamy, Priyanka Ghosh, Shweta Mahalingam, Priya Kadam, Harsha Prasada Lashkari, Parag Tamhankar, Vasundhara Tamhankar, Shilpa Mithbawkar, Sagar Bhattad, Prerna Jhawar, Adinarayan Makam, Vandana Bansal, Malathi Prasad, Geeta Govindaraj, Beena Guhan, Karthik Bharadwaj Tallapaka, Mukesh Desai, Revathi Raj, Manisha Rajan Madkaikar

**Affiliations:** ^1^Center of Excellence for PIDs, Department of Pediatric Immunology and Leucocyte Biology, ICMR-National Institute of Immunohaematology, Mumbai, India; ^2^Department of Pediatric Hematology-Oncology, Blood Marrow Transplantation, Apollo Hospitals, Chennai, India; ^3^Department of Clinical Genetics & Genetic Counseling, Mediscan Systems, Chennai, India; ^4^Department of Immunology and Department of Pediatric Hemato-Oncology, Bai Jerbai Wadia Hospital for Children, Mumbai, India; ^5^MedGenome Labs Private Limited, Bangalore, India; ^6^Department of Pediatrics, Kasturba Medical College Hospital, Manipal Academy of Higher Education, Mangalore, India; ^7^Centre for Medical Genetics, Mumbai, India; ^8^Division of Pediatric Immunology and Rheumatology, Department of Pediatrics, Aster CMI Hospital, Bangalore, India; ^9^Department of Fetal Medicine, Motherhood Hospital, Bangalore, India; ^10^Department of Fetal Medicine, Adi Advanced Centre for Fetal Care, Bangalore, India; ^11^Fetal Medicine Department Surya Hospitals, Mumbai, India; ^12^Fetal Medicine Centre, Trichy, India; ^13^Department of Pediatrics, Government Medical College, Kozhikode, Calicut, India; ^14^CSIR-Centre for Cellular and Molecular Biology (CSIR-CCMB), Hyderabad, India

**Keywords:** prenatal diagnosis, chorionic villus sampling, maternal contamination, cordocentesis, flow cytometry, variants of unknown significance

## Abstract

Prenatal Diagnosis (PND) forms an important part of primary preventive management for families having a child affected with primary immunodeficiency. Although individually sparse, collectively this group of genetic disorders represents a significant burden of disease. This paper discusses the prenatal services available for affected families at various centers across the country and the challenges and ethical considerations associated with genetic counseling. Mutation detection in the index case and analysis of chorionic villous sampling or amniocentesis remain the preferred procedures for PND and phenotypic analysis of cordocentesis sample is reserved for families with well-characterized index case seeking PND in the latter part of the second trimester of pregnancy. A total of 112 families were provided PND services in the last decade and the presence of an affected fetus was confirmed in 32 families. Post-test genetic counseling enabled the affected families to make an informed decision about the current pregnancy.

## Introduction

Primary immunodeficiency disorders (PIDs) are a heterogeneous group of single-gene disorders of the immune system with more than 450 distinct PIDs described in literature. The overall prevalence rate may be as high as 1:1,000 to 1:5,000 in the general population (1). Despite an improvement in the understanding of the molecular basis of PIDs, its optimum management remains a challenge. Hematopoietic stem cell transplantation (HSCT) from a HLA matched sibling is an option available only to about 30% of cases (2). Other forms of supportive therapy like regular intravenous immunoglobulin transfusions and prophylactic antibiotics especially for patients with X-linked Agammaglobulinemia (XLA) and Chronic Granulomatous Disease (CGD) may result in long-term survival but are associated with a poor quality of life and a prohibitive cost. This poses a significant financial and emotional burden on the family and society as a whole. Therefore, in families having a child affected with PID, genetic counseling and PND are the cornerstones of primary preventive management. This paper highlights the PND services for PID available in India, the different techniques utilized, and their comparison, the challenges, and ethical considerations.

## Materials and Methods

### Participant Details

We analyzed the PND services offered at eight centers across India to families with a known case of PID in the last decade. Clinical details of the index case, the underlying molecular defect in the index case, the technique used for PND, the result, and outcomes were studied.

### Sample Collection Techniques and Sample Processing

#### Chorionic Villus Sampling

In CVS, fetal chorionic tissue was obtained by aspiration either by transabdominal or trans cervical route at 11–13 weeks**’** gestation. After aspiration, fetal villi were dissected from the decidual tissue, and then genetic testing was performed on the DNA extracted from the same.

#### Amniocentesis

In amniocentesis, exfoliated fetal cells in amniotic fluid were studied for molecular analysis. This was performed at 16–18 weeks’ gestation. For samples collected by CVS or Amniocentesis, molecular analysis was performed by Sanger sequencing (for well-characterized mutation in the index case and parents) or Next generation sequencing. Maternal cell contamination (MCC) was ruled out by VNTR analysis.

#### Sampling of Fetal Blood or Cordocentesis

Cordocentesis was performed at 18–20 weeks of gestation in families in which the index case was immunophenotypically well-characterized and molecular analysis was not available. Also, situations where CVS or amniocentesis failed to provide a definite diagnosis, PND by cordocentesis was offered to affected families. The phenotypic evaluation comprised of lymphocyte subset analysis for Severe combined immunodeficiency (SCID), HLA-DR expression on lymphocytes for MHC class II deficiency, BTK expression on monocytes for XLA, CD18/CD11a integrin expression on leukocytes for Leukocyte adhesion defect (LAD1), and oxidative burst activity of fetal neutrophils by dihydrorhodamine assay for CGD. The interpretation of results was based on comparison with previously established reference ranges (3).

MCC was ruled out by Kleihauer–Betke test and confirmed by VNTR analysis. Before the aforementioned tests, another simple and rapid way for ruling out MCC in fetal blood was performed by checking high mean corpuscular volume (MCV) value (>110 fL) with a narrow, single red cell distribution curve before sample processing.

### Ethical Approval

The study is approved by the Institutional Ethics Committee of ICMR-National Institute of Immunohaematology.

## Result

**Figure 1:**
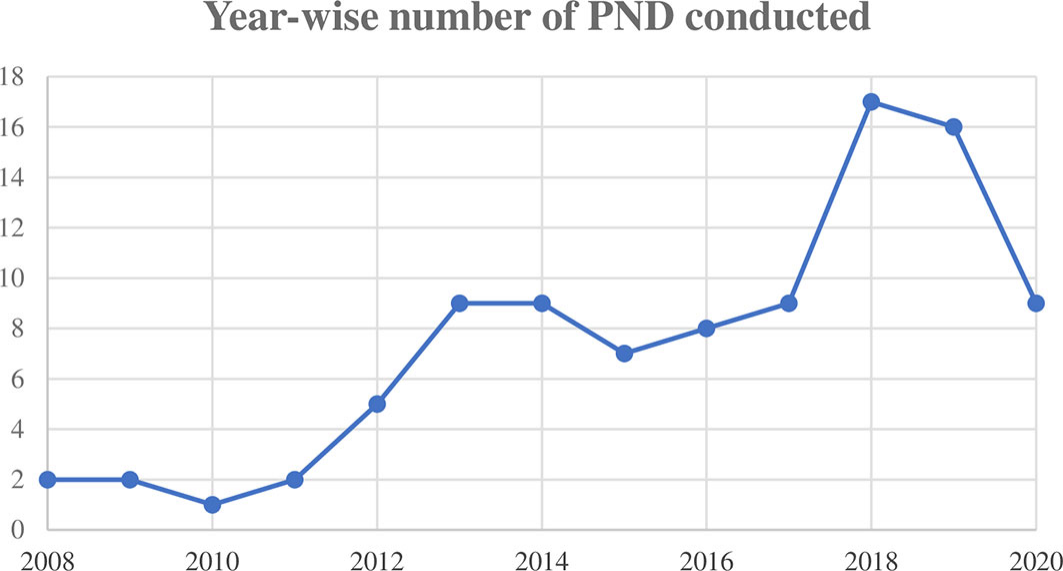
Year-wise number of prenatal diagnosis (PND) conducted.

Prenatal testing was performed in total of 112 affected families (n = 121 pregnancies). Of these, phenotypic prenatal diagnoses were performed in 44 and molecular analysis was performed in 77 cases. The year-wise number of prenatal tests conducted is depicted in **Figure 1**. Consanguinity was reported in 44% of the couples seeking PND.

**Figure 2:**
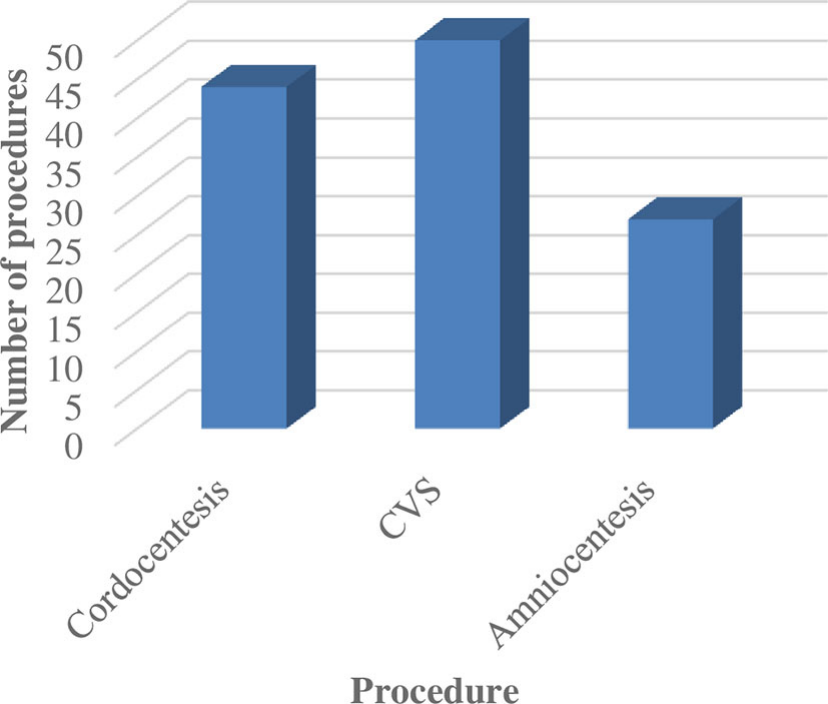
Comparing the application of different prenatal diagnostic techniques.

The prenatal diagnostic techniques used for PND are summarized in **Figure 2**.

**Figure 3:**
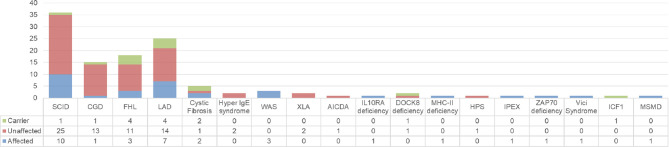
Summary of diagnosis in index case (n) and the result of PND (affected, unaffected, carrier).

Prenatal diagnosis was most commonly sought for SCID, LAD, FHL and CGD. The diagnosis in the index case (proband) and the results of the investigations in the index cases and prenatal cases are summarized in **Figure 3** (**Supplementary Table 1**). Seven of the 112 families seeking PND had variants of unknown significance (VUS) identified in index case and parents.

Diagnosis following cordocentesis was offered to 43/44 families. In one family with LAD1(P42) in the index case, the diagnosis could not be offered due to significant MCC. Cord blood samples after delivery confirmed the accuracy of diagnosis in all but one case of LAD1(P4) in the child, and she expired at two months of age. Procedure-related complications were observed in two families—one pregnancy was aborted due to infection and the second one due to rough travel soon after the cordocentesis procedure.

PND confirmed the presence of an affected fetus in 32 families, and 31/32 of these pregnancies were terminated; one family with a diagnosis of SCID decided to continue with the pregnancy, and it was confirmed after birth. Carrier status was detected in eight fetuses [one CGD family, one DOCK8 family, three LAD1, and one LAD3 family, one SCID family, and one Immunodeficiency, Centromeric instability, and Facial anomaly syndrome (ICF) family]; pregnancy was continued. In all cases with unaffected or carrier fetuses, the diagnosis was confirmed on cord blood samples after delivery and further follow-up of the children. Two such families are as yet on antenatal follow-up.

## Discussion

India has a significant collective burden of genetic disorders which can be attributed to a high rate of consanguinity, which is generally observed in approximately 14% marriages according to the National Family Health Survey (NFHS-4) report 2015–16 (4–6). In our cohort, consanguinity was reported by 44% of the total couples seeking PND. 33% of fetuses among consanguineous marriages were found to be affected compared to 22% affected fetuses in non-consanguineous marriages.

These services are commonly offered for various genetic disorders including hemoglobinopathies and Down’s syndrome in India. The rising trend of PND sought for PID over the last decade shows an increasing awareness about the availability of these services in affected families and about PIDs itself. PND services including molecular diagnoses are being offered by some genetic centers both in the public and private sectors.

CVS was the technique most commonly deployed for sampling in our cohort followed by cordocentesis and amniocentesis. Mutation detection in the index case and analysis of CVS or amniocentesis remain the preferred sampling procedures for PND due to high sensitivity and specificity and allow a sufficient window for safe termination of pregnancy wherever required depending on the test results (7). As far as the ethics, the psychological distress and the health risk to the mother are concerned; the earlier the screening is performed, the better. In addition to the timing factor, the risk to the mother or fetus of the invasive techniques and the accessibility or cost of the techniques are important factors for consideration (8). The most dreaded complication after these invasive sampling procedures is fetal loss. The incidence of fetal loss subsequent to the procedure is highest with cordocentesis (1.4%), and comparable between CVS (0.2%) and amniocentesis (0.3%). Lower fetal losses are documented for centers having experience in performing these procedures. A higher number of attempts, presence of fetal structural abnormalities, underlying placental disorders, uterine malformations, and fibroids are associated with increased chances of fetal loss following the procedure (9, 10). The high risk of fetal loss with cordocentesis as reported in literature was consistent with our observation of fetal loss after two cordocentesis procedures. However, since most couples in India report for prenatal testing in the latter part of the second trimester (11), a phenotypic diagnosis does have a role, and cordocentesis is reserved only for such families. Due to the higher risk of fetal loss associated with cordocentesis, there is a need to stress upon reporting early in the next pregnancy for PND when genetic counseling is offered after diagnosis in the index case.

Cordocentesis followed by phenotypic analysis using flow cytometry is a sensitive, reproducible, and rapid technique for PND especially for patients seeking prenatal testing beyond 18 weeks of gestation as the results can be made available within 24 h of the procedure. We have established at our center, reference ranges on cordocentesis samples at 18 weeks of gestation as described in a previous paper (3, 12). The technique has an added advantage in detecting an affected fetus in the situation of partial maternal contamination, thereby precluding the need for a repeat procedure. However, phenotypic analysis has a few limitations (13, 14) as observed in one LAD1 family where the child was found to be affected at birth. As such, confirming the surface expression of a molecule may be misleading as the protein expression though normal might have a defective function and specifically for LAD1, checking for expression of CD11a in addition to CD18 becomes important as it is consistently abnormal in all patients with LAD1, whereas CD18 expression may be variable or functionally abnormal despite normal expression.

The presence of MCC in fetal samples can lead to prenatal misdiagnosis and must be ruled out in all PND. American College of Medical Genetics and Clinical Molecular Genetics Society have laid standards and guidelines for cytogenetic and molecular genetic testing that recommend MCC testing in prenatal diagnosis. For cordocentesis, looking at Kleihauer–Betke test and MCV value are quick methods to rule out MCC although confirmation by variable number tandem repeat (VNTR) remains the gold standard technique (15).

Next generation sequencing is being increasingly offered for the detection of mutation during PND (16, 17) especially in cases where a mutation is not identified in the index case. Since mutations in the Indian population are not well characterized, many pathogenic variants also get labeled as VUS which presents a challenge for genetic counseling. In our cohort, seven such families who had sought PND had VUS identified in the index case and parents. Once a VUS is identified, it needs to be correlated with the phenotype in the index case. All efforts should be made that the decision is made with a full understanding of the findings and its clinical relevance (18).

Utilization of non-invasive prenatal diagnosis through circulating cell-free fetal DNA (cffDNA) analysis in maternal circulation (19, 20) is being explored as it nullifies the risk of miscarriages, reduces the cost of the procedure, and may be utilized early in gestation. cffDNA is highly fragmented in nature (fragments of length <300 bp) and smaller than maternally derived sequences and the levels increase with increasing gestation (21). The quantitation of cffDNA is subject to variation in the gestational age, delay in processing time, extraction method used, and sensitivity of the detection technique. Currently, NIPT is mainly used to detect aneuploidy, fetal Rh D typing, X-linked genetic diseases, and some single gene inheritance diseases including Thalassemia (22) and has limited application in PID.

The provision of PND services benefits the high-risk family in making a decision about the current pregnancy. In the situation where the family makes an informed decision to continue with the pregnancy even though it would lead to the birth of an affected child, knowing the genetic diagnosis can lead to early intervention after birth. Affected infants diagnosed with SCID as neonates may have a better survival due to timely HSCT than those in whom disease detection is delayed.

On one hand, information obtained through genetic testing can be empowering in reproductive decision making, while on the other hand, the influence of this knowledge may result in psychological harm, stigmatization, and discrimination (23). To enhance decision making by a patient, ethical considerations include pretest counseling which should provide accurate information about the testing procedure and the risks involved, including the possibility of ambiguous results. Both the medical and social consequences of the proposed tests and their results should be discussed (24). Genetic counseling also has an important bearing in revealing carrier status to a mother in case of diagnosis of an X-linked condition in the index case as it can seriously affect her status within the family. This raises important concerns regarding the rights of individual privacy and familial privacy, and what information others outside the family can have access to especially biological relatives who also might be affected (25). Ethical concerns regarding genetic counseling also become especially important in the event of medically actionable incidental findings in parents and VUS in the fetus (26). Another ethical concern in that a substantial proportion of people in certain communities do not consider abortion as a morally acceptable option even in face of an affected pregnancy.

This study highlights the increasing awareness about PIDs and the knowledge about the availability of PND services for preventing the birth of an affected child. However, the uptake of these services is not yet sufficient to address the needs of all affected families. Although these conditions are amenable to diagnosis in the first trimester by analysis of CVS specimens, at present most are diagnosed by analyzing fetal blood taken at 18–20 weeks’ gestation, and hence the need to counsel the affected families for seeking timely PND services.

## Data Availability Statement

The original contributions presented in the study are included in the article/**Supplementary Material**, further inquiries can be directed to the corresponding author.

## Ethics Statement

The studies involving human participants were reviewed and approved by ICMR-National Institute of Immunohaematology. Written informed consent to participate in this study was provided by the participants’ legal guardian/next of kin.

## Author Contributions

RMY compiled the data, wrote and edited the manuscript. Standardization of phenotypic analysis was done by MG, SSha, and AD. Molecular testing was done by GH, JA, MK, PKam, SSes, SJ, BS, JR, MS, PG, SMah, PKad, SMit, AM, KT. RR, RU, UB, PTau, HL, PTam, VT, SB, PJ, GG, BG, and MD provided the clinical details, provided genetic counseling, and followed up the patients. MM supervised the study, reviewed and approved the final version of the manuscript. All authors contributed to the article and approved the submitted version.

## Conflict of Interest

SMal, PG, SMah, and PKad were employed by the company MedGenome Labs Private Limited. SJ, BS, and JR were employed by the company Mediscan Systems.

The remaining authors declare that the research was conducted in the absence of any commercial or financial relationships that could be construed as a potential conflict of interest.

## References

[1] Tangye SG Al-Herz W Bousfiha A Chatila T Cunningham-Rundles C Etzioni A Human Inborn Errors of Immunity: 2019 Update on the Classification from the International Union of Immunological Societies Expert Committee. J Clin Immunol (2020) 40:24–64. 10.1007/s10875-019-00737-x 31953710 PMC7082301

[2] Uppuluri R Sivasankaran M Patel S Swaminathan VV Ramanan KM Ravichandran N Haploidentical Stem Cell Transplantation with Post-Transplant Cyclophosphamide for Primary Immune Deficiency Disorders in Children: Challenges and Outcome from a Tertiary Care Center in South India. J Clin Immunol (2019) 39:182–7. 10.1007/s10875-019-00600-z PMC7100782 30778805

[3] Mishra A Gupta M Dalvi A Ghosh K Madkaikar M Rapid Flow Cytometric Prenatal Diagnosis of Primary Immunodeficiency (PID) Disorders. J Clin Immunol (2014) 34:316–22. 10.1007/s10875-014-9993-7 24535004

[4] Soheilirad Z What should be the focus of counseling in parental consanguinity: genetic disorders or underlying beliefs. Egypt J Med Hum Genet (2020) 21:8. 10.1186/s43042-020-0049-7

[5] Sathyanarayana Rao T Asha M Sambamurthy K Jagannatha Rao K Consanguinity: Still a challenge. Indian J Psychiatry (2009) 51:3. 10.4103/0019-5545.44897 19742202 PMC2738413

[6] International Institute for Population Sciences (IIPS) and ICF National Family Health Survey (NFHS-4), 2015-16 (2017). India. Mumbai: IIPS Available at: http://rchiips.org/nfhs/NFHS-4Reports/India.pdf (Accessed September 26, 2020).

[7] Verma IC Noninvasive Prenatal Testing: The Indian Perspective. J Fetal Med (2014) 1:113–8. 10.1007/s40556-014-0025-8

[8] Salomon LJ Sotiriadis A Wulff CB Odibo A Akolekar R Risk of miscarriage following amniocentesis or chorionic villus sampling: systematic review of literature and updated meta-analysis. Ultrasound Obstet Gynecol (2016) 54:440–9. 10.1002/uog.20353 31124209

[9] Yenilmez ED Tuli A New Perspectives in Prenatal Diagnosis of Sickle Cell Anemia. In: Inusa BPD, editor. Sickle Cell Disease - Pain and Common Chronic Complications. InTech (2016). 10.5772/64646

[10] Ghi T Sotiriadis A Calda P Da Silva Costa F Raine-Fenning N Alfirevic Z ISUOG Practice Guidelines: invasive procedures for prenatal diagnosis in obstetrics. Ultrasound Obstet Gynecol (2016) 48:256–68. 10.1002/uog.15945 27485589

[11] Colah RB Gorakshakar AC Nadkarni AH Invasive & non-invasive approaches for prenatal diagnosis of haemoglobinopathies: Experiences from India. Indian J Med Res (2011) 134:552–60. PMC3237256 22089620

[12] Madkaikar MR Gupta M Rao M Ghosh K Prenatal Diagnosis of LAD-I on Cord Blood by Flowcytometry. Indian J Pediatr (2012) 79:1605–9. 10.1007/s12098-012-0737-5 22477041

[13] Sigmon JR Kasasbeh E Krishnaswamy G X-linked agammaglobulinemia diagnosed late in life: case report and review of the literature. Clin Mol Allergy (2008) 6:5. 10.1186/1476-7961-6-5 18518992 PMC2424073

[14] Delmonte OM Schuetz C Notarangelo LD RAG Deficiency: Two Genes, Many Diseases. J Clin Immunol (2018) 38:646–55. 10.1007/s10875-018-0537-4 PMC6643099 30046960

[15] Ghi T Sotiriadis A Calda P Da Silva Costa F Raine‐Fenning N Alfirevic Z International Society of Ultrasound in Obstetrics and Gynecology (ISUOG). ISUOG Practice Guidelines: invasive procedures for prenatal diagnosis. ISUOG (2016) 48:256–68. 10.1002/uog.15945 27485589

[16] Mellis R Chandler N Chitty LS Next-generation sequencing and the impact on prenatal diagnosis. Expert Rev Mol Diagn (2018) 18:689–99. 10.1080/14737159.2018.1493924 29962246

[17] Vora NL Hui L Next-generation sequencing and prenatal ‘omics: advanced diagnostics and new insights into human development. Genet Med (2018) 20:791–9. 10.1038/s41436-018-0087-4 PMC6123255 30032162

[18] Harris S Gilmore K Hardisty E Lyerly AD Vora NL Ethical and counseling challenges in prenatal exome sequencing. Prenatal Diagn (2018) 38:897–903. 10.1002/pd.5353 PMC6370459 30171820

[19] Massa JD Arora V Lallar M Bijarnia S Puri RD Verma IC Current Status of Noninvasive Prenatal Testing and Counselling Considerations: An Indian Perspective. J Fetal Med (2020) 7:9–16. 10.1007/s40556-019-00228-4

[20] Yang WC Zhu L Qiu YM Zhou BX Cheng JL Wei CL Isolation and analysis of cell-free fetal DNA from maternal peripheral blood in Chinese women. Genet Mol Res (2015) 14:18078–89. 10.4238/2015 December.22.34. 26782455

[21] Parchure DS Kulkarni SS Noninvasive fetal RHD genotyping from maternal plasma. Glob J Transfus Med (2016) 1:21–8. 10.4103/2455-8893.178007

[22] Kwun Chiu RW Dennis Lo YM Clinical applications of maternal plasma fetal DNA analysis: translating the fruits of 15 years of research. Clin Chem Lab Med (2013) 51:197–204. 10.1515/cclm-2012-0601 23072857

[23] Stapleton G Qualifying choice: ethical reflection on the scope of prenatal screening. Med Health Care Philos (2017) 20:195–205. 10.1007/s11019-016-9725-2 27631408 PMC5487727

[24] Darryl RJ Macer Ethics and Prenatal Diagnosis. Genetic Disorders and the Fetus: Diagnosis, Prevention and Treatment. Milunsky A, editor. Baltimore: John Hopkins University Press (1998). p. 999–1024.

[25] Gates EA Ethical considerations in prenatal diagnosis, In Fetal Medicine [Special Issue]. West J Med (1993) 159:391–5. PMC1011355 8236982

[26] Amendola LM Lautenbach D Scollon S Bernhardt B Biswas S East K Illustrative case studies in the return of exome and genome sequencing results. Personalized Med (2015) 12:283–95. 10.2217/pme.14.89 PMC4607287 26478737

