# Supplementary material for: Prenatal Diagnosis for Primary Immunodeficiency Disorders—An Overview of the Indian Scenario
Source: Front Immunol. 2020 Dec 7;11:612316. doi: 10.3389/fimmu.2020.612316 (PMC7750517; doi:10.3389/fimmu.2020.612316)
Supplement: Supplementary file 1 [file Table_1.docx]

| **Index case** | | | | | | **History of consanguinity** | **Carrier status** | | **PND** | | | | |
| --- | --- | --- | --- | --- | --- | --- | --- | --- | --- | --- | --- | --- | --- |
| **Proband ID** | **Diagnosis** | **Mutation details** | | | |  |  |  | **Procedure** | **Flowcytometry result** | **Result** | **Mutation identified**  **(if affected)** | **Outcome** |
|  |  | **Gene** | **Location** | **Variant** | **Zygosity** |  | **Mother** | **Father** |  |  |  |  |  |
| P1 | WAS | *WAS* | Exon 9 | c.823_834dup | Heterozygous | Yes | Carrier | ₋ | CVS | ND | Affected | *WAS* Exon 9 c.823_834dup | Terminated |
| P2 | SCID | *JAK3* | Exon 22 | c.3049_3050delCT | Homozygous | Yes | Carrier | Carrier | CVS | ND | Unaffected | No mutation identified | Doing well |
| P3 | XLA | *BTK* | Exon 3 | c.240G>T | Hemizygous | No | ₋ | ₋ | Amniocentesis | ND | Unaffected | No mutation identified | Doing well |
| P4 | LAD-I | Mutation not available | | | | Yes | ₋ | ₋ | Cordocentesis | CD18+ on 94% neutrophils. | Affected* | Not available | Girl baby delivered, died after 2 months of life |
| P5 | Hyper IgE | *STAT3* | Exon 21 | c.1970A>G | Heterozygous  (Autosomal dominant) | No | ₋ | ₋ | Amniocentesis | ND | Unaffected | No mutation identified | Doing well |
| P6 | CGD | *CYBB* |  | c.1234G>A | Heterozygous | No | Carrier | ₋ | CVS | ND | Unaffected | No mutation identified | Doing well |
| P7 | Hyper IgE | *STAT3* | Exon 10 | c.986T>A | Homozygous | No | Carrier | Affected | CVS | ND | Unaffected | No mutation identified | Doing well |
| P8 | SCID | No clinically relevant mutation found | | | | Yes | ₋ | ₋ | CVS | ND | Affected | Details not available | Terminated |
| P9 | SCID | *JAK3* | Details not available | | | No | ₋ | ₋ | CVS | ND | Unaffected | No mutation identified | Doing well |
| P10 | WAS | *WAS* | Exon 9 | c.919A>G | Heterozygous | No | Carrier | ₋ | CVS | ND | Affected | *WAS* Exon 9 c.919A>G | Terminated |
|  | WAS | *WAS* | Exon 10 | c.919A>G | Heterozygous | No | Carrier | ₋ | CVS | ND | Affected | *WAS* Exon 9 c.919A>G | Terminated |
| P11 | FHL | No clinically relevant mutation found | | | | No | ₋ | ₋ | CVS | ND | Unaffected | No mutation identified | Doing well |
| P12 | LAD-I | No clinically relevant mutation found | | | | No | ₋ | ₋ | Amniocentesis | ND | Unaffected | No mutation identified | On antenatal follow-up |
| P13 | SCID | *ADA* | Details not available | | | No | ₋ | ₋ | Amniocentesis | ND | Unaffected | No mutation identified | On antenatal follow-up |
| P14 | FHL | *UNC13D* | Exon 30 | c.2866C>T | Compound heterozygous | No | Carrier | Carrier | Amniocentesis | ND | Unaffected | No mutation identified | Doing well |
|  |  |  | Exon 31 | c.3053_3054delCC |  |  | ₋ | ₋ |  |  |  |  |  |
| P15 | AICDA | *AICDA* | Exon 2 | c.A64T | Homozygous | No | Carrier | Carrier | Amniocentesis | ND | Unaffected | No mutation identified | Doing well |
| P16 | FHL | *RAB27A* | Exon 6 | c.550C>T | Homozygous | No | Carrier | Carrier | CVS | ND | Unaffected | No mutation identified | Doing well |
| P17 | SCID | *RAG1* | Exon 2 | c.2849delT | Compound heterozygous | No | Carrier | Carrier | Amniocentesis | ND | Affected | *RAG1* c.2849delT | Terminated |
|  |  |  | Exon 2 | c.1421G>A |  |  |  |  |  |  |  | c.1421G>A |  |
| P18 | HPS | *HPS1* | Intron 5 | c.398+5G>A | Homozygous | No | Carrier | Carrier | Amniocentesis | ND | Unaffected | No mutation identified | Doing well |
| P19 | IL10RA deficiency | *IL10RA* | Exon 4 | c.493C>T | Homozygous | No | Carrier | Carrier | Amniocentesis | ND | Affected | *IL10RA* c.493C>T | Terminated |
| P20 | CGD | *NCF1* | Exon 2 | c.75_76delGT | Homozygous | No | Carrier | Carrier | Cordocentesis | 90% DHR Positive | Unaffected | ND | Doing well |
| P21 | CGD | No clinically relevant mutation found | | | | No | ₋ | ₋ | Cordocentesis | 99% DHR Positive | Unaffected | ND | Doing well |
| P22 | CGD | *CYBB* | Exon 7 | c.771_777del  CCCAATC | Heterozygous | No | Normal | Normal | Cordocentesis | 70% DHR positive | Unaffected | ND | Doing well |
| P23 | CGD | No clinically relevant mutation found | | | | No | ₋ | ₋ | Cordocentesis | 79% DHR Positive | Unaffected | ND | Doing well |
| P24 | CGD | No clinically relevant mutation found | | | | No | ₋ | ₋ | Cordocentesis | 91% DHR Positive | Unaffected | ND | Doing well |
| P25 | CGD | No clinically relevant mutation found | | | | No | ₋ | ₋ | Cordocentesis | 58% DHR Positive | Unaffected | ND | Doing well |
| P26 | CGD | *NCF2* | Exon 12 | c.1148_1149TGdel | Homozygous | Yes | Carrier | Carrier | CVS | ND | Unaffected | No mutation identified | Doing well |
| P27 | CGD | *NCF2* | Exon 5 | c.550C>T | Homozygous | Yes | ₋ | ₋ | Cordocentesis | 73% DHR Positive | Unaffected | ND | Doing well |
| P28 | CGD | *NCF1* | Details not available | | | No | ₋ | ₋ | Cordocentesis | 84% DHR Positive | Unaffected | ND | Doing well |
| P29 | CGD | No clinically relevant mutation found | | | | No | ₋ | ₋ | Cordocentesis | Absent DHR | Affected | ND | Terminated |
| P30 | CGD | *CYBB* | Exon 11 | c.1426G>A | Homozygous | No | Carrier | ₋ | CVS | ND | Carrier | *CYBB* Exon 11 c.1426G>A | Doing well |
| P31 | CGD | *CYBB* | Exon 3 | c.217C>T | Heterozygous | No | ₋ | ₋ | CVS | ND | Unaffected | No mutation identified | Doing well |
| P32 | CGD | *NCF1* | Exon 2 | c.75_76delGT | Homozygous | Yes | Carrier | Carrier | Cordocentesis | 81% DHR Positive | Unaffected | ND | Doing well |
| P33 | FHL | *PRF1* | Exon 2 | c.528_529 delinsAA | Homozygous | No | Carrier | Carrier | CVS | ND | Unaffected | No mutation identified | Doing well |
|  |  |  |  |  |  |  | Carrier | Carrier | CVS | ND | Affected | *PRF1* c.528_529delinsAA | Terminated |
| P34 | FHL | *PRF1* | Exon 2 | c.386G>C | Homozygous | Yes | Carrier | Carrier | CVS | ND | Unaffected | *PRF1* c.386G>C(Het) | Doing well |
|  |  |  |  |  |  |  | Carrier | Carrier | CVS | ND | Carrier | *PRF1* c.386G>C p.Y129S(Het) | Doing well |
| P35 | FHL | *PRF1* | Exon 2 | c.528_529 delinsAA | Homozygous | No | Carrier | Carrier | CVS | ND | Affected | *PRF1* c.528_529delinsAA | Terminated |
|  |  |  |  |  |  |  | Carrier | Carrier | CVS | ND | Affected | *PRF1* c.528_529delinsAA | Terminated |
| P36 | FHL | *UNC13D* | Exon 29 | c.2722insACCT | Homozygous | No | Carrier | Carrier | CVS | ND | Carrier | *UNC13D* c.2772 ins ACCT (Het) | Doing well |
| P37 | FHL | *STX11* | Exon 2 | c.404T>C | Heterozygous | No | Carrier | Carrier | CVS | ND | Carrier | *STX 11* c.404T>C (Het) | Doing well |
|  |  |  |  |  |  |  | Carrier | Carrier | CVS | ND | Unaffected | No mutation identified | Doing well |
| P38 | FHL | *PRF1* | Exon 3 | c.148G>A | Homozygous | Yes | Carrier | Carrier | CVS | ND | Unaffected | No mutation identified | Doing well |
| P39 | FHL | *PRF1* | Exon 2 | c.386 G>C | Homozygous | No | Carrier | Carrier | CVS | ND | Carrier | *PRF1* c.386 G>C (Het) | Doing well |
| P40 | LAD - I | *ITGB2* | Exon 12 | c.1632C>G, c.1828C>A | Compound heterozygous | No | Carrier | Carrier | Cordocentesis | ND | Unaffected | No mutation identified | Doing well |
|  |  |  |  |  |  |  |  |  | CVS | ND | Unaffected | No mutation identified | Doing well |
|  |  |  |  |  |  |  |  |  | Cordocentesis | CD18% CD11 0.2% | Affected | ND | Terminated |
| P41 | LAD - I | *ITGB2* | Exon 9 | c.1057_1057delinsG c.1059_1059delinsA | Homozygous | Yes | Carrier | Carrier | Cordocentesis | CD18% CD11 97% | Unaffected | No mutation identified | Doing well |
| P42 | LAD - I | *ITGB2* | Details not available | | | No | ₋ | ₋ | Cordocentesis |  | MCC |  |  |
| P43 | LAD - I | *ITGB2* | Exon 9 | c.1057_1057delinsG c.1059_1059delinsA | Homozygous | No | Carrier | Carrier | Cordocentesis | CD18% CD11 97% | Unaffected | ND | Doing well |
| P44 | LAD - I | *ITGB2* | Details not available | | | Yes | ₋ | ₋ | Cordocentesis | CD18% CD11 0.2% | Affected | ND | Terminated |
| P45 | LAD - I | *ITGB2* | Exon 13 | c.1777C>T | Homozygous | No | ₋ | ₋ | CVS |  | Affected | *ITGB2* Exon 13 c.1777C>T | Terminated |
| P46 | LAD - I | *ITGB2* | splice site | c.817(G-A), IVS 7+1 (G-C) | Compound heterozygous | No | ₋ | ₋ | CVS |  | Affected | *ITGB2* c.817(G-A); IVS 7+1 (G-C)-Compound heterozygous | Terminated |
| P47 | LAD - I | *ITGB2* | Exon 6 | c.533c>T | Homozygous | No | ₋ | ₋ | Cordocentesis | CD18% CD11 95% | Unaffected | ND | Doing well |
| P48 | LAD - I | *ITGB2* | Exon 7 | c.850G>A | Homozygous | No | Carrier | Carrier | Cordocentesis | CD18% CD11 97% | Unaffected | ND | Doing well |
| P49 | LAD - I | *ITGB2* | Exon 7 | c.850G>A | Homozygous | No | ₋ | ₋ | Amniocentesis | ND | Unaffected | No mutation identified | Doing well |
| P50 | LAD - I | *ITGB2* | Exon 7, Intron 7 | c.897+1(G-C); IVS7+1(G-C),817G>A | Heterozygous | No | ₋ | ₋ | CVS | ND | Carrier | *ITGB2* splice site mutation c.897+1(G-C); IVS7+1(G-C) | Doing well |
| P51 | LAD - I | *ITGB2* | Exon 7 | c.817G>A | Homozygous | yes | Carrier | Carrier | CVS | ND | Affected | *ITGB2* Exon 7 c.817G>A | Terminated |
| P52 | LAD - I | *ITGB2* | Exon 6 | c.725A>G | Homozygous | Yes | Carrier | Carrier | CVS | ND | Carrier | *ITGB2* Exon 6 c.725A>G | Doing well |
| P53 | LAD - I | *ITGB2* | Exon 6 | c.505G>A | Homozygous | Yes | ₋ | Carrier | CVS | ND | Carrier | *ITGB2* Exon 6 c.505G>A | Doing well |
| P54 | LAD - I | *ITGB2* | Exon 7 | c.817G>A | Homozygous | Yes | Carrier | Carrier | CVS | ND | Carrier | No mutation found | Doing well |
| P55 | LAD - I | *ITGB2* | Exon 4 | c.322C>T | Homozygous | Yes | Carrier | Carrier | Cordocentesis | CD18 0% CD11 0% | Affected | *ITGB2* c.322C>T | Terminated |
| P56 | LAD - I | *ITGB2* | Exon 7 | c.817G>A | Homozygous | No | Carrier | Carrier | Cordocentesis | CD18 100% CD11 100% | Unaffected | ND | Doing well |
| P57 | MHC-II deficiency | *RFXAP (+)* | Exon 1 | c.460_461insC | Homozygous | No | ₋ | ₋ | Cordocentesis | 67% of B cells did not show HLA-DR expression | Affected | ND | Terminated |
| P58 | SCID | Details not available | | | | No | ₋ | ₋ | Cordocentesis | Absence of T and NK cells | Affected | ND | Terminated |
| P59 | SCID | Details not available | | | | No | ₋ | ₋ | Cordocentesis | Absence of T and B cells | Affected | ND | Terminated |
| P60 | SCID | Details not available | | | | yes | ₋ | ₋ | Cordocentesis | Normal percentage of lymphocytes | Unaffected | ND | Doing well |
| P61 | SCID | *RAG1* | Exon 2 | c.2146C>T | Homozygous | yes | ₋ | ₋ | Cordocentesis | Normal percentage of lymphocytes | Unaffected | ND | Doing well |
| P62 | SCID | Details not available | | | | yes | ₋ | ₋ | Cordocentesis | Normal percentage of lymphocytes | Unaffected | ND | Doing well |
| P63 | SCID | Details not available | | | | No | ₋ | ₋ | Cordocentesis | Normal percentage of lymphocytes | Unaffected | ND | Doing well |
| P64 | SCID | Details not available | | | | No | ₋ | ₋ | Cordocentesis | Normal percentage of lymphocytes | Unaffected | ND | Doing well |
| P65 | SCID | Details not available | | | | No | ₋ | ₋ | Cordocentesis | Normal percentage of T cells, NK % lower | Unaffected | ND | Doing well |
| P66 | SCID | Details not available | | | | No | ₋ | ₋ | Cordocentesis | Normal percentage of lymphocytes | Unaffected | ND | Doing well |
| P67 | SCID | Details not available | | | | No | ₋ | ₋ | Cordocentesis | Normal percentage of lymphocytes | Unaffected | ND | Doing well |
| P68 | ZAP70 deficiency | ZAP70 | Exon 3 | c.183 T>A | Homozygous | Yes | ₋ | ₋ | CVS |  | Affected | *ZAP70* c.183 T>A | Terminated |
| P69 | SCID | Details not available | | | | Yes | ₋ | ₋ | CVS | Normal percentage of lymphocytes | Unaffected | ND | Doing well |
|  |  |  |  |  |  |  | ₋ | ₋ | CVS |  | Carrier | Mutation not available | Doing well |
| P70 | XLA | Details not available | | | | No | ₋ | ₋ | Cordocentesis | 12% Bcells Btk on CD14+ Monocytes 86% | Unaffected | ND | Doing well |
| P71 | DOCK8 deficiency | *DOCK8* | Exon 36 | c.4346C>A | Homozygous | Yes | ₋ | ₋ | Amniocentesis | ND | Carrier | *DOCK8* c.4346C>A | NA |
| P72 | Cystic Fibrosis | *CFTR* | Exon 20 | c.3209G>A | Heterozygous | Yes | Carrier | Carrier | CVS | ND | Carrier | *CFTR* c.3209G>A | NA |
| P73 | ICF1 | *DNMT3B* | Exon 23 | c.2452G>T | Homozygous | Yes | Carrier | Carrier | CVS | ND | Carrier | *DNMT3B* c.2452G>T | NA |
| P74 | Cystic Fibrosis | *CFTR* | Exon 25 | c.4110_4111insGAA(VUS) , c.1520_1522del | Compound heterozygous | No | ₋ | ₋ | CVS | ND | Carrier | *CFTR* c.4110_4111insGAA (VUS) | NA |
|  | IPEX | *FOXP3* | Exon 12 | c.1249C>T | Homozygous | Yes | ₋ | ₋ | CVS | ND | Affected | *IPEX c.1249C>T* | Terminated |
| P75 | Cystic Fibrosis | *CFTR* | chr7 | NA | Compound heterozygous | No | Carrier | Carrier | CVS | ND | Affected | *CFTR* c.53+1G>T (5’ splice site) and c.3484C>T | Terminated |
| P76 | Cystic Fibrosis | *CFTR* | chr7 | NA | Homozygous | Yes | ₋ | ₋ | Amniocentesis | ND | Affected | *CFTR* c.53+1G>T | Terminated |
| P77 | Vici Syndrome | *EPGS* | Exon 2 | c.519A>T , c.6311C>G(VUS) | Compound heterozygous | Yes | ₋ | ₋ | Amniocentesis | ND | Affected | *EPGS* c.519A>T,c.6311C>G(VUS) | Terminated |
| P78 | Cystic Fibrosis | *CFTR* | Exon 5 | c.547C>A - VUS, c.6311C>G-VUS,c.2811G>A | Homozygous | No | ₋ | ₋ | Amniocentesis | ND | Unaffected | No mutation identified | NA |
| P79 | Ataxia pancyto-  penia syndrome | *SAMD9L* | Exon 5 | c.1934T>A(VUS) | Homozygous | No | ₋ | ₋ | Amniocentesis | ND | Maternal contamination |  | NA |
| P80 | ALPS | *FADD* | Exon 2 | c.315T>G(VUS), parents carrier of this mutation | Heterozygous | Yes | Carrier | Carrier | CVS | ND | VUS | *FADD* c.315T>G(VUS) | NA |
| P81 | DOCK8 deficiency | *DOCK8* | Exon 28 | c.3220C>A(VUS) | Heterozygous | Yes | ₋ | ₋ | Amniocentesis | ND | VUS | *DOCK8* c.3220C>A(VUS) | NA |
| P82 | ATM | *ATM* | Exon 10 | c.1295T>A(VUS) | Heterozygous | Yes | Carrier | Carrier | Amniocentesis | ND | VUS | *ATM* c.1295T>A(VUS) | NA |
| P83 | DOCK8 deficiency | *DOCK8* | Exon 35 | c.4346C>A | Heterozygous | Yes | ₋ | ₋ | Amniocentesis | ND | Unaffected | No mutation identified | Doing well |
| P84 | LAD-III | *FERMT3* | Exon 12 | c.1343G>A | Heterozygous | No | ₋ | ₋ | CVS | ND | Unaffected | No mutation identified | Doing well |
| P85 | LAD-I | *ITGB2* | Exon 6 | c.533C>T | Homozygous | No | ₋ | ₋ | Amniocentesis | ND | Unaffected | No mutation identified | Doing well |
| P86 | LAD-I | *ITGB2* | Details not available | | | Yes | ₋ | ₋ | Amniocentesis | ND | Unaffected | No mutation identified | Doing well |
| P87 | FHL | *PRF1* | Exon 2 | c.386G>C | Homozygous | Yes | ₋ | ₋ | Amniocentesis | ND | Unaffected | No mutation identified | Doing well |
| P88 | LAD-I | *ITGB2* | Exon 6 | missense variant | Homozygous | Yes | ₋ | ₋ | CVS | ND | Unaffected | No mutation identified | Doing well |
| P89 | SCID | *JAK3* | Exon 11 | c.1442A>G | Homozygous | Yes | ₋ | ₋ | CVS | ND | Unaffected | No mutation identified | Doing well |
| P90 | SCID | *IL2RG* | Exon 5 | c.664C>T | Hemizygous | No | ₋ | ₋ | Amniocentesis | ND | Unaffected | No mutation identified | Doing well |
| P91 | SCID | *CD40LG* | Exon 3 | c.308-309delAG | Hemizygous | No | ₋ | ₋ | Amniocentesis | ND | Affected | *CD40L*c.308-309delAG | Terminated |
| P92 | SCID | *JAK3* | Exon 13 | c.1717G>C | Homozygous | Yes | ₋ | ₋ | Amniocentesis | ND | Affected | *JAK3*c.1717G>C | Terminated |
| P93 | SCID | *ZBTB24* | Exon 4 | c.1204+2 T>A | Homozygous | Yes | ₋ | ₋ | CVS | ND | Unaffected | No mutation identified | Doing well |
| P94 | SCID | *ADA* | Exon 11 | c.986C>T | Homozygous | Yes | ₋ | ₋ | Amniocentesis | ND | Affected | *ADA*c.986C>T | Terminated |
| P95 | SCID | Details not available | | | | No | ₋ | ₋ | Cordocentesis | Normal percentage of lymphocytes | Unaffected | ND | Doing well |
| P96 | SCID | Details not available | | | | Yes | ₋ | ₋ | Cordocentesis | Normal percentage of lymphocytes | Unaffected | ND | Doing well |
| P97 | SCID | *IL2RG* | Details not available | | | No | ₋ | ₋ | CVS | ND | Unaffected | No mutation identified | Doing well |
| P98 | SCID | Details not available | | | | Yes | ₋ | ₋ | Cordocentesis | Normal percentage of lymphocytes | Unaffected | ND | Doing well |
| P99 | FHL | *UNC13D* |  | c.118-308C>T | Homozygous | No | ₋ | ₋ | CVS | ND | Unaffected | No mutation identified | Doing well |
| P100 | CGD | *CYBB* | Exon 7 |  | Hemizygous | No | ₋ | ₋ | CVS | ND | Unaffected | No mutation identified | Doing well |
| P101 | SCID | Mutation not available | | |  | Yes | ₋ | ₋ | Cordocentesis | Normal percentage of lymphocytes | Unaffected | ND | Doing well |
| P102 | SCID | *JAK3* | Details not available | |  | Yes | ₋ | ₋ | Cordocentesis | Markedly reduced T cells | Affected | JAK3 | Terminated |
| P103 | SCID | Mutation not available | | |  | Yes | ₋ | ₋ | Cordocentesis | Normal percentage of lymphocytes | Unaffected | ND | Doing well |
| P104 | SCID | Mutation not available | | |  | Yes | ₋ | ₋ | Cordocentesis | Normal percentage of lymphocytes | Unaffected | ND | Doing well |
| P105 | MSMD. | *IFNGR1* | Exon 7 | c.1068delG | Homozygous | Yes | ₋ | ₋ | CVS |  | Affected | *IFNGR1*c.1068delG | Terminated |
| P106 | SCID | Mutation not available | | |  | No | ₋ | ₋ | Cordocentesis | Normal percentage of lymphocytes | Unaffected | ND | Doing well |
| P107 | SCID | Mutation not available | | |  | Yes | ₋ | ₋ | Cordocentesis | Markedly reduced T cells | Affected | ND | Terminated |
| P108 | SCID | *JAK3* |  |  |  | No | ₋ | ₋ | Cordocentesis | Markedly reduced T cells | Affected | ND | Terminated |
| P109 | FHL | *STXBP2* | Exon 19 | c.1730G>A | Homozygous | No | ₋ | ₋ | CVS | ND | Unaffected | No mutation identified | Doing well |
| P110 | SCID | *JAK3* | Exon 13 | c.1744C>T | Homozygous | Yes | ₋ | ₋ | Amniocentesis | ND | Unaffected | No mutation identified | Doing well |
| P111 | FHL | *PRF1* | Exon 2 | c.386G>C | Heterozygous | Yes | ₋ | ₋ | Amniocentesis | ND | Unaffected | No mutation identified | Doing well |
| P112 | SCID | Mutation not available | | |  |  | ₋ | ₋ | Cordocentesis | Normal percentage of lymphocytes | Unaffected | ND | Doing well |
